# Overall Vertical Transmission of Hepatitis C Virus, Transmission Net of Clearance, and Timing of Transmission

**DOI:** 10.1093/cid/ciac270

**Published:** 2022-04-11

**Authors:** Anthony E Ades, Fabiana Gordon, Karen Scott, Intira J Collins, Thorne Claire, Lucy Pembrey, Elizabeth Chappell, Eugènia Mariné-Barjoan, Karina Butler, Giuseppe Indolfi, Diana M Gibb, Ali Judd

**Affiliations:** Population Health Sciences, University of Bristol Medical School, Bristol, United Kingdom; Population Health Sciences, University of Bristol Medical School, Bristol, United Kingdom; Medical Research Council Clinical Trials Unit, University College London, London, United Kingdom; Medical Research Council Clinical Trials Unit, University College London, London, United Kingdom; Population, Policy and Practice Research and Teaching Department, UCL Great Ormond Street Institute of Child Health, London, United Kingdom; Department of Medical Statistics, Faculty of Epidemiology and Population Health, London School of Hygiene and Tropical Medicine, London, United Kingdom; Medical Research Council Clinical Trials Unit, University College London, London, United Kingdom; Université Côte d’Azur, Public Health Department, Centre Hospitalier Universitaire de Nice, Nice, France; Children's Health Ireland at Crumlin and Temple Street, Dublin, Ireland; Meyer Children's Hospital and Department Neurofarba, University of Florence, Firenze, Italy; Medical Research Council Clinical Trials Unit, University College London, London, United Kingdom; Medical Research Council Clinical Trials Unit, University College London, London, United Kingdom

**Keywords:** hepatitis C virus, HCV, vertical transmission, spontaneous clearance, net transmission

## Abstract

**Background:**

It is widely accepted that the risk of hepatitis C virus (HCV) vertical transmission (VT) is 5%–6% in monoinfected women, and that 25%–40% of HCV infection clears spontaneously within 5 years. However, there is no consensus on how VT rates should be estimated, and there is a lack of information on VT rates “net” of clearance.

**Methods:**

We reanalyzed data on 1749 children in 3 prospective cohorts to obtain coherent estimates of overall VT rate and VT rates net of clearance at different ages. Clearance rates were used to impute the proportion of uninfected children who had been infected and then cleared before testing negative. The proportion of transmission early in utero, late in utero, and at delivery was estimated from data on the proportion of HCV RNA positive within 3 days of birth, and differences between elective cesarean and nonelective cesarean deliveries.

**Results:**

Overall VT rates were 7.2% (95% credible interval [CrI], 5.6%–8.9%) in mothers who were human immunodeficiency virus (HIV) negative and 12.1% (95% CrI, 8.6%–16.8%) in HIV-coinfected women. The corresponding rates net of clearance at 5 years were 2.4% (95% CrI, 1.1%–4.1%), and 4.1% (95% CrI, 1.7%–7.3%). We estimated that 24.8% (95% CrI, 12.1%–40.8%) of infections occur early in utero, 66.0% (95% CrI, 42.5%–83.3%) later in utero, and 9.3% (95% CrI, 0.5%–30.6%) during delivery.

**Conclusions:**

Overall VT rates are about 24% higher than previously assumed, but the risk of infection persisting beyond age 5 years is about 38% lower. The results can inform design of trials of interventions to prevent or treat pediatric HCV infection, and strategies to manage children exposed in utero.

## Teaser

Taking account of infections that would have cleared spontaneously before detection, the rate of hepatitis C virus vertical transmission is 7.2% in monoinfected women, but transmission “net” of clearance is 3.1% at 3 years and 2.4% at 5 years.

**(See the Major Article by Ades et al on pages 913–9 and the Editorial Commentary by Jhaveri on pages 920–2.)**

With the discovery of direct-acting antivirals to treat hepatitis C virus (HCV), attention is turning to interventions either in pregnancy or in infancy to prevent or treat vertically acquired infection. The World Health Organization's target of HCV elimination by 2030 [1] has added further urgency to this issue. According to a 2014 meta-analysis, vertical transmission (VT) occurs in 5.8% of infants of HCV RNA–positive mothers who are not human immunodeficiency virus (HIV) coinfected, and 10.8% if mothers also have HIV [2]. A proportion of vertically infected infants clear spontaneously by age 5 years: 20%–40% is cited in reviews and guidelines [3, 4], but a recent analysis reported 66% clearance, with rates initially high then declining over the first 3 years [5].

This pattern of clearance means that the VT rates reported in the literature depend on the age at which infection status is ascertained and also on the timing of diagnostic tests. The lack of standardization in testing schedules and in methods for calculating transmission rates has long been a cause for concern [6, 7]. Some studies have included all children meeting the definition of infection even if they subsequently clear, while others do not; some report outcomes at 18 months. Each strategy will produce a different estimate of the VT rate.

A second problem is that some infections may clear before they are detected and confirmed. An infant whose first RNA test is at 3 months and is negative would be counted as uninfected in a prospective study, but they may have been infected and then cleared before 3 months. If the first negative RNA test was at 6 months, an initial infection would have had longer in which to clear, and the probability that the child had originally been infected would be correspondingly greater.

The likelihood that unobserved infection and clearance are occurring alongside the variation in how detected infections are counted introduces a profound lack of clarity about how to interpret the reported VT rates.

This article aims to give a coherent account of the underlying VT rate and the VT rate net of clearance at different ages. This is needed to inform strategies for prevention, diagnosis, and treatment of vertically acquired infection, and to plan trials of preventive and therapeutic interventions.

We use data on individual mother-child pairs from 3 published European cohorts to estimate, for the first time, both the overall rate of confirmed VT and the VT rates net of clearance at ages up to 5 years. The overall (underlying) VT rate is estimated by correcting for infections that may have cleared before they were detected. VT rates net of clearance are then estimated by applying clearance rates, estimated previously from the same data [5], to the overall VT rate.

Our analysis also looks at the impact of mother's HCV RNA viral load, mother's HIV coinfection, and mode of delivery. We investigate the timing and mechanism of infection, by estimating the proportion of infection that occurs early in utero, later in utero, and during delivery. This may help inform the optimal timing of preventive treatment in pregnancy.

## METHODS

### Data Sources

Three prospective studies following infants born to HCV antibody (anti-HCV)–positive mothers were included: European Pediatric HCV Network (EPHN) [4, 8–10]; the British Paediatric Surveillance Unit (BPSU) study, which included 3 hospitals in Dublin, Irish Republic and centers across the United Kingdom [11]; and the ALHICE (Alpes-Maritimes, Languedoc, Haute Garonne Infection C chez l’Enfant) study [12]. The selection of these studies has been described previously [5], along with details of their pediatric testing schedules. The Faculty of Health Sciences Research Ethics Committee, University of Bristol, approved these analyses of historic data.

### Definitions

Infants were regarded as infected if they were ever anti-HCV positive after 18 months and/or had at least 2 positive RNA tests at any age. Those who did not meet the infected definition were considered uninfected if they tested RNA negative at any age after 6 weeks or if their final anti-HCV test was negative. Remaining children were considered “indeterminate.” Note that “infected” is to be interpreted as “ever-infected” because infected infants can subsequently clear infection, and that “uninfected” infants may have been infected and cleared. Supporting details are given in the Supplementary Materials.

Ages at which tests are performed play a key role in the estimation of the probability that each indeterminate infant was infected and that each uninfected infant had been infected, then cleared:

- Age at last anti-HCV positive under 18 months: The later the last positive anti-HCV test, the more likely the infant is to be infected.
- Age at last RNA negative under 6 weeks: The later this is, the less likely the infant is to have been infected.
- Age at first anti-HCV–negative test or the first negative RNA test over 6 weeks, whichever is earliest, is the age when the infant is first known to be uninfected: The later this is, the more likely the infant is to have been infected and cleared.

### Statistical Methods

Our objective was to estimate the risk of vertical infection, the impact of risk factors (mother's HIV and HCV RNA viral load), and the proportions of infection transmitted early in utero, late in utero, and at delivery. The proportion transmitted early in utero is informed directly by the proportion HCV RNA positive in the first 3 days. Assuming that children delivered by elective cesarean (ECS) cannot acquire infection during delivery, the difference between overall transmission rates in ECS and non-ECS modes of delivery informs the proportion of non–early in utero transmission that is late in utero as opposed to occurring during delivery, among those not delivered by ECS.

Infection at each stage, early in utero, late in utero, or during delivery, is conditional on not being infected at an earlier stage. Data are available on risk factors (study: EPHN, BPSU, ALHICE; mother's HIV status; and mother's HCV viral load measured as near as possible to delivery: low, high [>600 copies/mL]). Risk factors impact on risk of transmission in each of the 3 routes as they would in a standard logistic regression, but it is assumed that the odds ratios are the same for each route. We assumed that the log odds ratio associated with higher viral load could depend on HIV status. This interaction was constrained so that the log risk attaching to mothers’ positive HIV status and high HCV viral load combined had to be no less than the log risk of either factor alone, but could not be more than both added together. Standard interaction and main effect models were investigated as sensitivity analyses. All models controlled for study effects.

Mother-child pairs lacking data either on mode of delivery or mother's HIV status, and cases where the mother was known to be HCV RNA negative were excluded. Mother's HCV RNA infection status was unknown in 67% of the remaining records, and where RNA status was known to be positive, HCV viral load was unknown in 43% (Table 1). We included data with missing HCV RNA on the assumption that the proportions of mothers with low viral load, or no detectable RNA, were exactly the same as in mothers in the EPHN study with the same HIV status and mode of delivery whose HCV RNA status was known. Robustness of conclusions to these assumptions was assessed in sensitivity analyses assuming that the odds of both no HCV RNA and of low viral load were both either 1.6 times higher or 1.6 times lower, which we considered implausibly extreme.

**Table 1.** Infection Status and Risk Factor Distribution in the 3 Cohorts, After Removal of Records With Missing Human Immunodeficiency Virus, Missing Mode of Delivery, and RNA-Negative Mothers

Characteristic	EPHN		BPSU		ALHICE		Total	
	No.	(%)	No.	(%)	No.	(%)	No.	(%)
Total	1256	(100)	342	(100)	151	(100)	1749	(100)
Infection status								
ȃInfected	69	(5.5)	15	(4.4)	12	(7.9)	96	(5.5)
ȃIndeterminate	121	(9.6)	102	(29.8)	0	(0.0)	223	(12.8)
ȃUninfected	1066	(84.9)	225	(65.8)	139	(92.1)	1430	(81.8)
Mother’s HIV status								
ȃNo	1053	(83.8)	321	(93.1)	105	(69.5)	1479	(84.6)
ȃYes	203	(16.2)	21	(6.9)	46	(30.5)	270	(15.4)
Mother’s HCV viral load								
ȃLow	167	(13.3)	0	(0.0)	94	(62.3)	261	(14.9)
ȃHigh	29	(2.3)	0	(0.0)	39	(25.8)	68	(3.9)
ȃNK but RNA positive	240	(19.1)	0	(0.0)	4	(2.6)	244	(14.0)
ȃRNA NK	820	(65.3)	342	(100)	14	(9.3)	1176	(67.2)
Mode of delivery								
ȃECS	373	(29.7)	26	(3.3)	35	(23.2)	434	(24.8)
ȃNon-ECS	883	(70.3)	316	(96.7)	116	(76.8)	1315	(75.2)

Abbreviations: ALHICE, Alpes-Maritimes, Languedoc, Haute Garonne Infection C chez l’Enfant; BPSU, British Paediatric Surveillance Unit; ECS, elective cesarean section; EPHN, European Pediatric HCV Network; HCV, hepatitis C virus; HIV, human immunodeficiency virus; NK, not known.

In outline, the statistical analysis estimates the probability that each child of indeterminate status is infected, taking into account their risk group, the age when they were last anti-HCV positive, and the age at the last HCV RNA negative if this was <6 weeks. Similarly, the probability that each uninfected child was originally infected and then cleared is calculated, based again on risk group, and on the age when they were first ascertained as uninfected. These probability calculations are shown in Supplementary Table 1. The estimated probabilities of infection in each indeterminate and uninfected child are then summed and added to the number of children with confirmed infection to estimate a notional transmission rate. Uninfected children who were originally infected but then cleared are thus “restored” to the underlying overall VT rate. Then, the net VT rates at selected ages are estimated by applying the clearance rate to the overall VT rate. The statistical analysis was carried out using Bayesian Markov chain Monte Carlo estimation. Details of the statistical methods are given in the Supplementary Materials.

## RESULTS

The proportions infected, indeterminate, and uninfected and the risk factor distributions are shown in Table 1.

### Numbers of Ever-Infected Children

**Figure 1.**
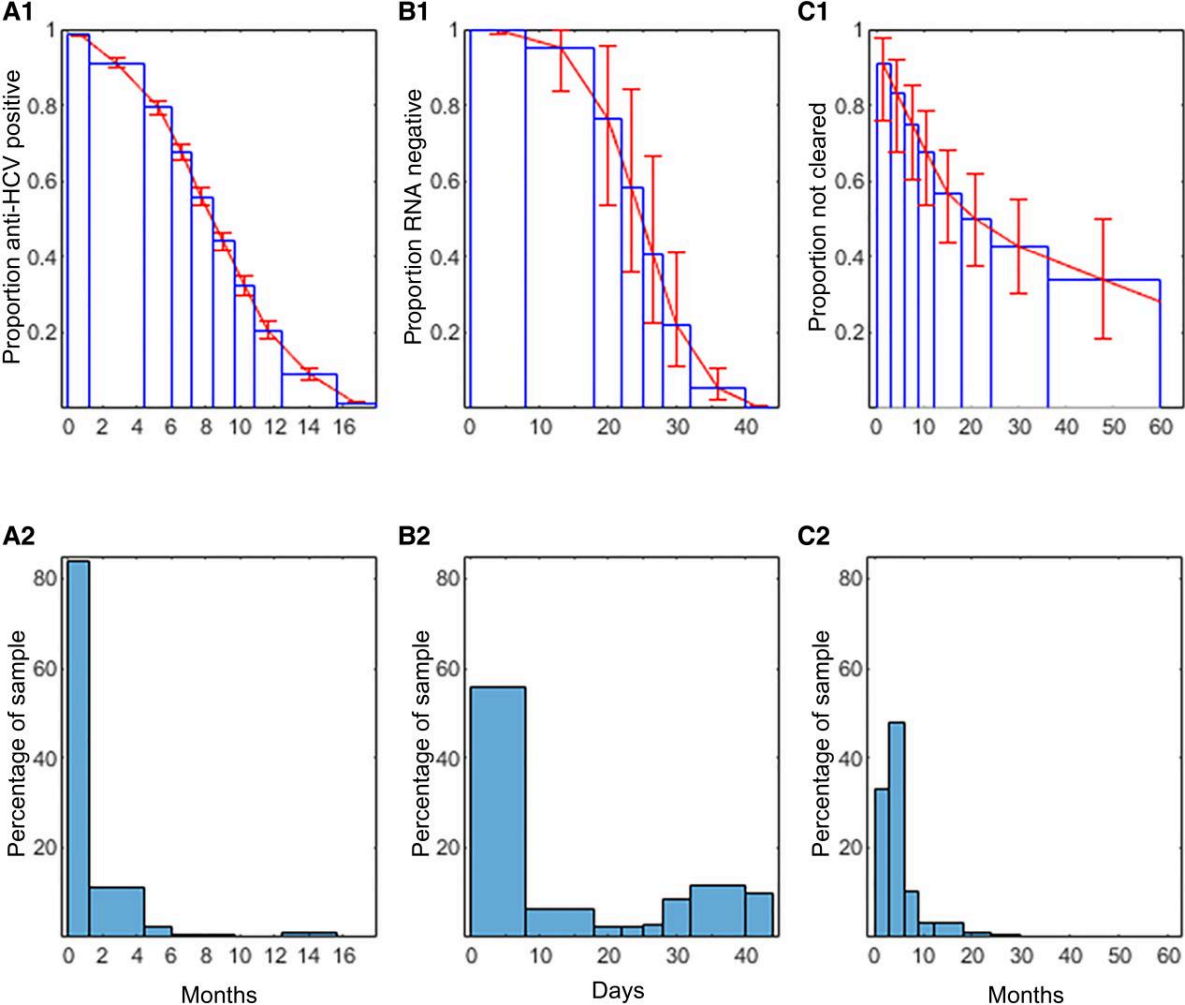
*A1*, Assumed proportion of uninfected children remaining hepatitis C virus antibody (anti-HCV) positive at each age up to 18 months. *B1*, Assumed proportion of infected children who are not initially RNA positive remaining RNA negative by age up to 6 weeks. *C1*, Proportion of infection not yet cleared, by age up to 5 years. *A2*, Proportion of indeterminates with last anti-HCV positive at each age. *B2*, Proportion of uninfected and indeterminate with last RNA negative at each age (<6 weeks). *C2*, Proportion of uninfected children with the first test indicating they were uninfected at each age.

Figure 1 *A1* shows the probability that uninfected children were anti-HCV positive by age; *B1* the probability that infected children were RNA negative under 6 weeks of age; *C1* the probability that an infected child had not cleared by age. These functions had been estimated from the 3 cohorts in advance, and are used together with the information in panels *A2*, *B2*, and *C2* to estimate the probability that individual uninfected and indeterminate children are infected. Figure 1*A2* is a histogram showing age at last positive anti-HCV among children with indeterminate status; *B2* shows age at last RNA negative under 6 weeks in uninfected and indeterminate children; *C2* shows age at first RNA or anti-HCV negative among uninfected children. The mean age when uninfected children of monoinfected women were first known to be uninfected was 5.2 months, and 4.4 months in HIV-coinfected women.

Table 2 illustrates the results of imputing the probability of infection in each indeterminate and uninfected child. In addition to the 96 observed infections, there were a further estimated 10.2 infections among the 223 children with indeterminate status, and a further 9.0 unobserved infections among the 1430 nominally uninfected infants, representing 8.6% and 7.8%, respectively, of the total 115.2 infections. In the entire combined cohort of 1749, the nominal VT rate is 6.6% (95 CrI%, 6.2%–7.1%) (Table 2). This is in a study population that includes 67% mothers who were anti-HCV positive but with unknown HCV RNA status, a proportion of whom—probably around 30%—would have been RNA negative and would not have transmitted.

**Table 2.** Observed and Unobserved Infections and Nominal Overall Vertical Transmission Rates

Infections	Infected	Indeterminate	Uninfected	Total
Totals	96	223	1430	1749
Observed infections	96	…	…	115.2 (108.7–124.2)
Unobserved infections	…	10.2 (7.1–13.8)	9.0 (4.3–17.1)	
Vertical transmission rate, %	…	4.7 (3.2–6.3)	0.6 (.3–1.2)	6.6 (6.2–7.1)

Data are presented as posterior mean (95% credible interval).

### Risk Factors and Timing of Transmission

Analysis of risk factors (Table 3) suggests no important differences between studies, and strong effects of both maternal HIV status and maternal HCV RNA viral load. Also shown are the absolute risks of transmission at each stage: early in utero, late in utero, and at delivery, in the HIV-negative low HCV RNA viral load group. The proportion of transmission by each route (Table 4) indicates that in non-ECS deliveries, 24.8%, 66.0%, and 9.3% of transmissions occur early in utero, late in utero, and at delivery. Among ECS deliveries we estimated 27.5% early and 72.5% late in utero. However, relatively few infected children, only 25, were tested in the first 3 days, of whom 9 (36%) tested positive, contributing to the wide credible intervals (CrIs) in estimated proportion of infection transmitted at delivery.

**Table 3.** Risk of Vertical Transmission by Route, and Odds Ratios for Study and Risk Group

Route and Risk Group	Posterior Median	Credible Interval	
		2.5%	97.5%
Risk of transmission, %, by route			
ȃEarly in utero	1.38	.64	2.59
ȃLate in utero	3.88	2.24	5.87
ȃDelivery	0.38	.03	1.97
Odds ratios, by study			
ȃEPHN	1 (ref)	…	…
ȃBPSU	1.23	.64	2.20
ȃALHICE	0.98	.47	1.87
Odds ratios, by risk group			
ȃHIV^−^, low VL	1 (ref)	…	…
ȃHIV^−^, high VL	2.66	1.19	6.12
ȃHIV^+^, low VL	1.75	1.08	3.12
ȃHIV^+^, high VL	3.43	1.69	8.03

Abbreviations: ALHICE, Alpes-Maritimes, Languedoc, Haute Garonne Infection C chez l’Enfant; BPSU, British Paediatric Surveillance Unit; EPHN, European Pediatric HCV Network; HIV, human immunodeficiency virus; VL, viral load.

**Table 4.** Percentage of Vertical Infection by Stage

Stage	Posterior Mean (95% Credible Interval)	
	Elective Cesarean	Nonelective Cesarean
Early in utero	27.5 (13.3–45.8)	24.8 (12.1–40.8)
Late in utero	72.5 (54.2–86.7)	66.0 (42.5–83.3)
At delivery	…	9.3 (.5–30.6)

**Figure 2.**
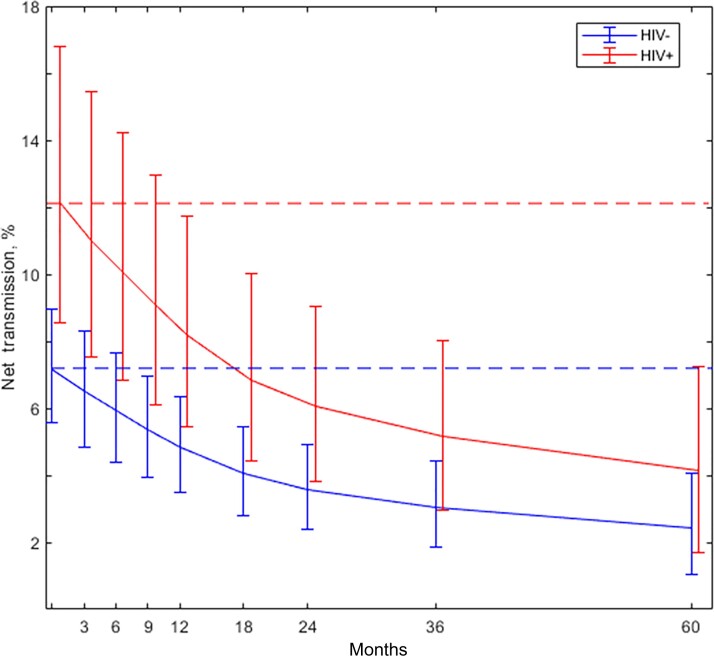
Overall vertical transmission (horizontal lines) and vertical transmission net of clearance at different ages: by mother's human immunodeficiency virus (HIV) status and weighted average of HIV negative (HIV^–^) and HIV positive (HIV^+^).

The overall VT rates by maternal HIV status, HCV viral load, and mode of delivery are shown in Table 5, and the average net VT rates at ages from 3 months to 5 years are plotted in Figure 2 separately for children of monoinfected and HIV-coinfected mothers. In these groups overall transmission risks are 7.2% and 12.1%, respectively, falling to VT rates net of clearance at 5 years of 2.4% (95%CrI, 1.1%–4.1%) and 4.1% (95% CrI, 1.7%–7.3%).

**Table 5.** Overall Vertical Transmission Rates, by Subgroup

Mode of Delivery	HCV Viral Load	HIV Negative			HIV Positive		
		Mean	Lower (2.5%) Credible Limit	Upper (97.5%) Credible Limit	Mean	Lower (2.5%) Credible Limit	Upper (97.5%) Credible Limit
ECS	Low	5.6	3.7	7.6	9.6	5.6	14.8
	High	14.2	7.1	23.5	17.5	10.2	27.7
Non-ECS	Low	6.1	4.2	8.2	10.6	6.2	16.2
	High	15.3	8.1	24.6	19.1	11.4	29.8
Weighted average		7.2	5.6	8.9	12.1	8.6	16.8

Abbreviations: ECS, elective cesarean section; Mean, posterior mean; HCV, hepatitis C virus; HIV, human immunodeficiency virus.

### Sensitivity Analyses

Sensitivity analyses (Table 6) suggest that the overall VT rates and the proportion of infection by each route are relatively insensitive to how or whether the impact of HCV RNA on transmission depends on HIV status, and to assumptions about the distribution of HCV RNA (high or low viral load, or negative) in data where this information was missing. Goodness of fit statistics fail to distinguish between the alternative models (a difference of <3 is not regarded as meaningful), and none of the variations in modeling assumptions raise or lower key estimates by >5%, well within the statistical uncertainty of the preferred model.

**Table 6.** Sensitivity Analyses

Model	Goodness of Fit	Overall VT, %, HIV Negative	Overall VT, %, HIV Positive	Transmission by Stage, %	
Preferred model					
*ȃ*Constrained interaction	741.6	7.2	12.1	24.8	9.3
Statistical uncertainty in preferred model					
*ȃ*Lower (2.5%) credible limit	…	5.6	8.6	12.1	0.5
*ȃ*Upper (97.5%) credible limit	…	8.9	16.8	40.8	30.6
Model choice					
ȃMain effect model	742.4	7.2	11.8	24.6	9.3
ȃSimple interaction model	743.0	7.2	11.8	24.6	9.2
Proportion low HCV viral load and proportion RNA negative					
ȃBoth odds lower by a factor of 1.6	742.2	7.4	12.2	24.6	9.1
ȃBoth odds higher by a factor of 1.6	740.9	7.0	12.1	24.8	9.4

Comparison of preferred model and alternatives. The preferred model is constrained interaction and assumes that 68.9% of the HIV negative and 90.7% of the HIV positive with unknown HCV RNA status are RNA positive. Goodness of fit is posterior mean deviance.

Abbreviations: HCV, hepatitis C virus; HIV, human immunodeficiency virus; VT, vertical transmission.

## DISCUSSION

HCV vertical transmission rates reported in the literature are based on infection status assessed at different ages, with no consensus on how to take account of spontaneous clearance. We have therefore developed an approach that estimates how many uninfected children may have been infected and cleared before their infection was detected and confirmed, based on a previously estimated clearance rate [5], and which then calculates VT rates net of clearance at ages from birth to age 5 years.

**Figure 3.**
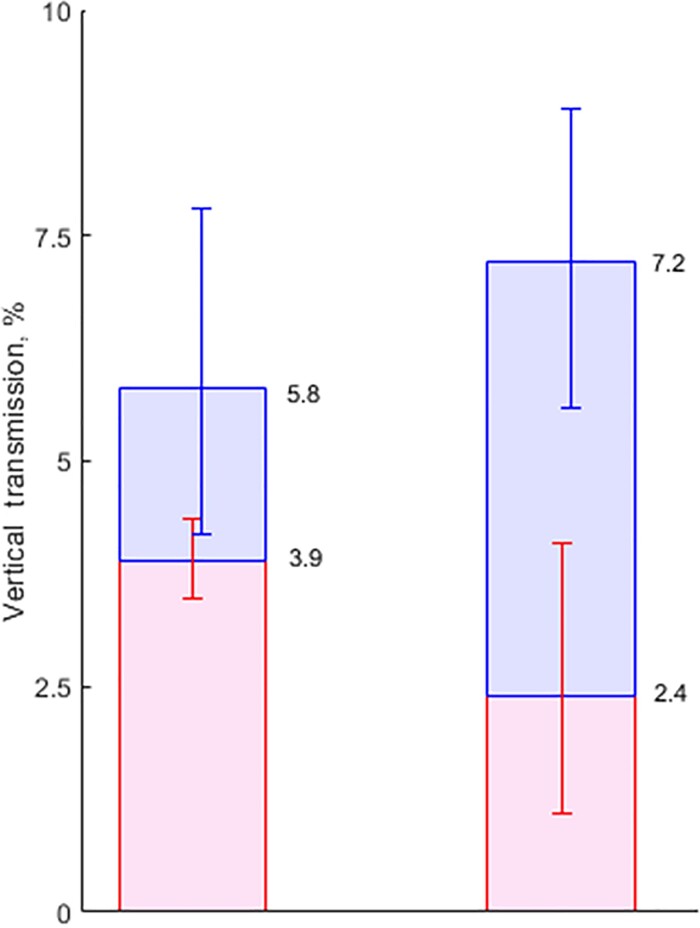
Left bar: Vertical transmission (VT) rate of 5.8% (95% credible interval [CrI], 4.2%–7.8%) in hepatitis C virus–monoinfected women [2], and spontaneous clearance of 32.5% (25%–40%) [14, 15]. Right bar: This study, with VT rate of 7.2% (95% CrI, 5.6%–8.9%) with 65.1% clearance (95%CrI, 50.1%–81.6%). Blue segments: infection that clears within 5 years. Red segments: infection remaining after 5 years.

When comparing results to previous literature, it is useful to consider VT rates in HIV-uninfected and HIV-coinfected mothers separately. The most recent meta-analysis of VT rates [2] reports 5.8% VT in HIV-negative women. If we now apply 25%–40% clearance rates [13] (average 32.5%) to this, we would predict that 3.9% of infants born to HCV RNA–positive mothers remain infected at 5 years. These figures can be compared to our estimated 7.2% overall transmission in monoinfected women and 2.4% net transmission at age 5 years. Thus, according to our analysis, the extent of VT is 24% higher than the accepted estimate, while the extent of chronic infection remaining at 5 years is 38% lower. Credible intervals should of course be taken into account (Figure 3).

As a “reality check,” we may note that the meta-analysis VT rate of 5.8% [2] is 81% of our estimate of 7.2%. If we refer this to the time-to-clearance curve [5], we find that this would represent a VT rate net of clearance at just under 6.8 months, which accords closely with the average age at which uninfected children were first known to be uninfected, 5.2 months. A similar exercise in HIV-coinfected women would show that the meta-analytic estimate of 10.8% represents a VT rate net of clearance at 3.6 months given our 12.1% overall VT rate; this compares to the 4.4-month average age at which children of HIV-infected mothers were first known to be uninfected.

The analysis has a number of limitations. Much of the data was collected at a time when polymerase chain reaction (PCR) tests were less accurate: various estimates of sensitivity and specificity of the tests used during this period have been made [5, 16, 17], but, like most investigators, we have taken test results at face value for the sake of simplicity. This may have impacted on the classification of children as infected, uninfected, and indeterminate, on the assumed time to loss of anti-HCV in uninfected infants, time to clearance, and time to positive RNA in infected infants.

Our estimate of the VT rate in HIV-coinfected women, 12.1%, may be of little contemporary relevance. The majority of coinfected women would have been treated with the less potent antiretroviral drugs available up to 2003. More recent European cohort studies including HIV/HCV-coinfected women with a high coverage of antiretroviral therapy suggest substantially lower HCV VT rates, in the range of 2.8%–5.9% [18–20].

A further important drawback is the extent of missing data on mother's viral load and HCV RNA status. Although sensitivity analyses reveal that results are robust against large changes in the assumed proportions RNA negative or with low viral load, this lack of data has prevented us from investigating whether HIV and HCV RNA status might impact transmission differently in utero or at delivery, or on clearance rates themselves. These questions do not appear to have been investigated previously, but can be researched within the framework we have introduced.

Finally, one can question whether the timing and frequency of tests in our cohorts was sufficient for accurate estimation of VT and clearance rates. In conventional analyses less frequent testing will impact on the numbers counted as infected or uninfected. By contrast, in our analyses less frequent testing will translate into greater statistical uncertainty in estimated time to clearance, which is then reflected in the credible intervals on overall and net VT rates. In theory, our methods should estimate the same clearance and VT rates that would be observed if children were tested every day, regardless of testing intervals. How close it comes to this ideal depends on sample size, with larger numbers needed if testing is less frequent. It is therefore relevant to note that although there was insufficient testing in the first 3 days, the intensity of subsequent testing was comparable to what would be expected in a well-conducted study today: The median age at the first HCV RNA test in the entire cohort was 4 days in the 88% who were ever tested; the median times between successive tests after that (whether antibody or HCV RNA) were 2.8, 3.1, and 3.8 months respectively.

A major contribution of this work is that it introduces methodology for simultaneously estimating overall VT rates and rates net of clearance. The novel element is the imputation of previously cleared infections among uninfected children, based on the age at which they were first known to be uninfected. This extends similar methodology used to impute the number of infections among indeterminates, both in the present study and in earlier studies of HCV [11] and HIV, before PCR testing became widely available [21–23].

The second contribution is the findings on VT net of clearance, which may help inform the design of trials of treatments in pregnancy to prevent vertical transmission, despite the shortcomings in the data. A recent phase 1 trial has been completed [24] and further trials are under way [25, 26]. Currently, the recommended care of children exposed in utero is to delay diagnosis until 18 months and then refer anti-HCV positives for RNA confirmatory testing at 3 years prior to treatment [3]. This strategy may not be viable where there is substantial loss to follow-up, as has been reported in infants born to HCV-infected women in the United States [27–30]. Our results may therefore also be relevant to evaluate alternative diagnostic and pediatric treatment strategies if and when treatments are licensed for use in children aged <3 years.

## Supplementary Data

Supplementary materials are available at *Clinical Infectious Diseases* online. Consisting of data provided by the authors to benefit the reader, the posted materials are not copyedited and are the sole responsibility of the authors, so questions or comments should be addressed to the corresponding author.

## Supplementary Material

ciac270_Supplementary_Data

## Notes

***Author Contributions.*** A. E. A., with the assistance of F. G., conceived and carried out the analyses. A. E. A. wrote the first and subsequent drafts of the paper. K. S. carried out the literature search and review. A. E. A., A. J., I. J. C., and D. M. G. were co-investigators on the Partnership to cure hepatitis C virus (HCV) in mono- and HIV/HCV co-infected pregnant women and prevent VERTical HCV transmission (HCVAVERT) project, and A. J. was the principal investigator. E. C. was a researcher on the HCVAVERT project. L. P., E. M.-B., D. M. G., and K. B. were senior or principal investigators on the 3 contributing studies: EPHN (European Pediatric HCV Network), ALHICE (Alpes-Maritimes, Languedoc, Haute Garonne Infection C chez l’Enfant), and BPSU (British Paediatric Surveillance Unit). G. I. provided clinical input on hepatology and management of pediatric hepatitis C virus (HCV). Curation of the original data files available to the project was the responsibility of L. P. and C. T. (EPHN), D. M. G. and K. B. (BPSU), and E. M.-B. (ALHICE). Subsequent data processing was done by F. G. and A. E. A. All authors critically reviewed and revised drafts as necessary and approved the final version for submission.

***Acknowledgments.*** The authors thank all those who have contributed to the EPHN, BPSU, and ALHICE studies.

***Disclaimer.*** The funding sources did not have any influence on study design, data collection, analysis and interpretation of the data, writing of the report, or the decision to submit for publication.

***Financial support.*** This work was supported by the Medical Research Council (UK Research and Innovation) (MR/R019746/1), through the Joint Global Health Trials scheme. Work at the Great Ormond Street Institute of Child Health was supported by the National Institute for Health and Care Research Biomedical Research Centre, Great Ormond Street Hospital.
